# Chronic hearing loss turns out being a calcified chondroid mesenchymal neoplasm with FN1::FGFR2 fusion

**DOI:** 10.1007/s00405-024-09024-x

**Published:** 2024-10-15

**Authors:** Leonard Bauerschmitz, Abbas Agaimy, Markus Eckstein, Matthias Balk, Heinrich Iro, Stephan Schleder, Sven-Martin Schlaffer, Antoniu-Oreste Gostian

**Affiliations:** 1 Universitätsklinikum Erlangen, Hals-Nasen-Ohren-Klinik, Kopf- und Halschirurgie, Erlangen, Germany; 2 Universitätsklinikum Erlangen, Pathologisches Institut, Erlangen, Germany; 3 Barmherzige Brüder Klinikum St. Elisabeth Straubing, Diagnostische und interventionelle Radiologie, Straubing, Germany; 4 Universitätsklinikum Erlangen, Neurochirurgische Klinik, Erlangen, Germany; 5 Barmherzige Brüder Klinikum St. Elisabeth Straubing, Klinik für Hals-Nasen-Ohren-Heilkunde mit Kopf-Hals- und plastischer Gesichtschirurgie, Straubing, Germany

**Keywords:** Skull base tumor, Calcified chondroid mesenchymal neoplasm, CCMN, FGFR1, FGFR2, FN1, TEK, Chondroma, Chordoma, Chondrosarcoma, Temporomandibular Joint, TruSight-RNA-Fusion panel, PMT, Tenosynovial giant cell tumor, Ossifying fibromyxoid tumor, Chondrobastoma, Calcifying aponeurotic fibroma, FGF23, Phosphaturic mesenchymal tumors

## Abstract

A 53 year old female presented with a six-year history of right-sided slow deterioration in hearing and a feeling of pressure in the right ear. The patient had not experienced any pain but reported some paresthesia of the right half of the tongue, whereas no further other cranial nerve deficits were evident. The otoscopy was unremarkable as well as the rest of the clinical ENT examination except for a slight asymptomatic swelling of the right cheek. Imaging findings showed an expansive tumor infiltrating and destroying the right lateral skull base. The tumor was partially composed of cystic/regressive lesions with high contrast media uptake. The tumor had high-signal intensity with water-sensitive sequences (T2w) and was hypointense on T1w images. We performed a tumor resection via a transparotideal-infratemporal approach. Histologically, the tumor was composed of granular variably calcified chondroid matrix with extensive regressive changes and granulation-like tissue reaction associated with calcinosis and crystal deposition. Molecular analysis of the tumor via the TruSight- RNA-Fusion panel detected a fusion involving FN1::FGFR2, consistent with “calcified chondroid mesenchymal neoplasm” (CCMN), a rare tumor entity recently defined by Liu et al 2021. In regular follow-up care no residual tumor has been detected in imaging studies (MRI and CT) within 2 years and 4 months. The biology and consequently the radio sensitivity cannot be defined precisely since long term results are missing due to the first description of this entity in 2021. As a consequence, surgical resection is recommended as the treatment of choice. Thorough clinical and radiological follow-up is mandatory as local recurrences are to be expected due to the infiltrative behavior. In case of a loco regional recurrence the fusion with FGFR2 may represent a therapeutic option for a targeted therapy on molecular level.

## Introduction

Tumors in the infratemporal region are generally rare. Most common neoplasms of this region are chondromas, chordomas, chondrosarcomas and osteosarcomas [1–7]. The proximity of tumors of the lateral skull-base and the neurovascular bundle makes complete or radical resection challenging or mostly impossible [2, 8, 9].

Recently another rare tumor entity has been defined as “calcified chondroid mesenchymal neoplasm” (CCMN) by Liu et al. and reports on these tumors are scarce so far [10–13]. These tumors are characterized by fusions of Fibronectin-1 (FN1) and Receptor Tyrosine Kinases (RTKs) like Fibroblast Growth Factor Receptor 1 (FGFR1), FGFR2 and Tunica Interna Endothelial Cell Kinase (TEK). These lesions usually affect the temporomandibular joint (TMJ) region and are characterized by an infiltrating growth [10, 13]. The affection of the TMJ-region could be related to the involvement of FGFR2 in the mandibular condylar development [14]. Mass, swelling or pain are the main presenting symptoms [11]. Up to now there are only a few reported cases of CCMNs in the TMJ region with FN1 rearrangement within this already extremely rare tumors [10–13].

Because of overlapping radiological and especially histopathological findings of CCMNs and other chondroid neoplasms, correct diagnosis is a major challenge [10].

## Case presentation

### Medical history and diagnostic workup

A 53 year old female presented with a six-year history of right-sided slow deterioration in hearing and a feeling of pressure in the right ear.

Since initial intravenous treatment with cortison (250 mg sodium (prednisolone-21-succinate)) had not achieved any improvement, the patient underwent a CT scan of the head at another ENT-department. Radiologically, suspected inflammatory changes in the middle ear and a tympanic effusion were found, therefore a tympanostomy tube was inserted.

At presentation in our department no tinnitus, vertigo or otorrhea were reported or observed. The patient had not experienced any pain but reported some paresthesia of the right half of the tongue, whereas no further other cranial nerve deficits were evident. The audiogram showed a mixed hearing loss on the right side. The otoscopy was unremarkable as well as the rest of the clinical ENT examination except for a slight asymptomatic swelling of the right cheek.

### Imaging

**Fig. 1:**
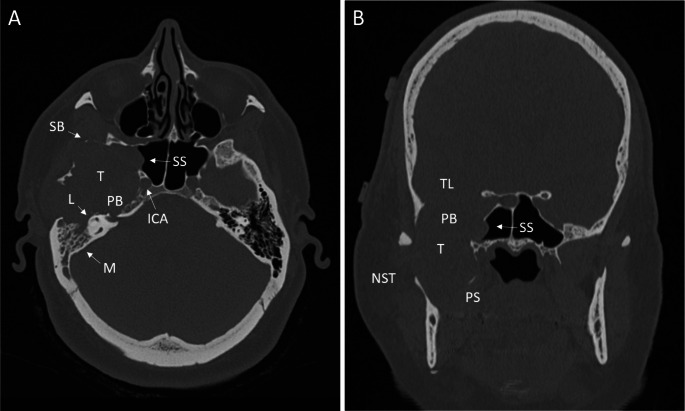
**CT scan.** A Axial and B coronar reconstruction. T: Tumor, SB: Destruction of the sphenoid bone, L: Labyrinth, PB: Destruction of the petrosal bone, M: Occluded mastoid, SS: Infiltration of the lateral wall of the sphenoid sinus, ICA: Internal carotid artery, PS: Infiltration of the parapharyngeal space, TL: Temporal lobe and invasion of the middle fossa, NST: Asymmetry of the neck soft tissue

**Fig. 2:**
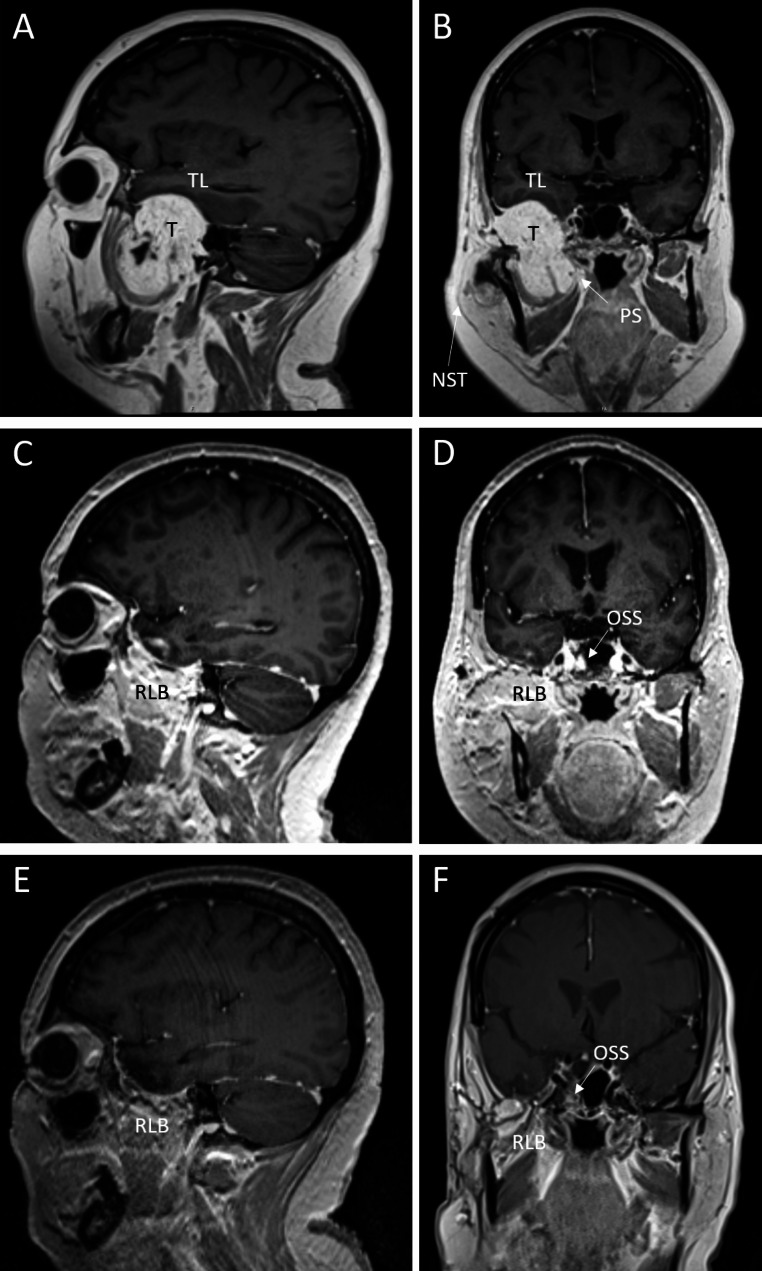
**MRI scan.** T1 with contrast, A and B: tumor extension preoperatively. Tumor (T) infiltrating the temporal and infratemporal fossa, parapharyngeal space (PS) and the middle fossa. Mild upwards compression of the right temporal lobe (TL). Asymmetry of the neck soft tissue (NST). C, D: 4 months postoperatively and E, F: 2 years and 4 months postoperatively no evidence of residual tumor and recurrence after reconstruction of the laterobase (RLB) and partial occlusion of the sphenoid sinus (OSS) with autologous fat. A, C, E: sagittal. B, D and F: coronar

CT and MRI scans of the head and neck region with and without contrast were acquired, respectively. Imaging findings showed an expansive tumor infiltrating and destroying the right lateral skull base (Fig. 1). The petrous bone and the mandibular joint were also destroyed. The tumor was partially composed of cystic/regressive lesions with high contrast media uptake (Fig. 2).

In total, the tumor had an extension of 5,8 × 5,3 × 4,7 cm.

The outer part of the right parotid gland, the right middle- and inner ear, the lateral wall of the sphenoid sinus, the temporal and infratemporal fossa and the carotid canal were tumor-infiltrated. Because of the tumor size there was a mild upwards compression of the right temporal lobe and an occluded tympanum and mastoid. T2w images showed fluid obstruction of the middle ear and mastoid cells on the right and T1w images prior to contrast agent administration showed partial signal-enhanced lesions, which may represent hemorrhage. The tumor had high-signal intensity with water-sensitive sequences (T2w) and was hypointense on T1w images.

### Treatment

**Fig. 3:**
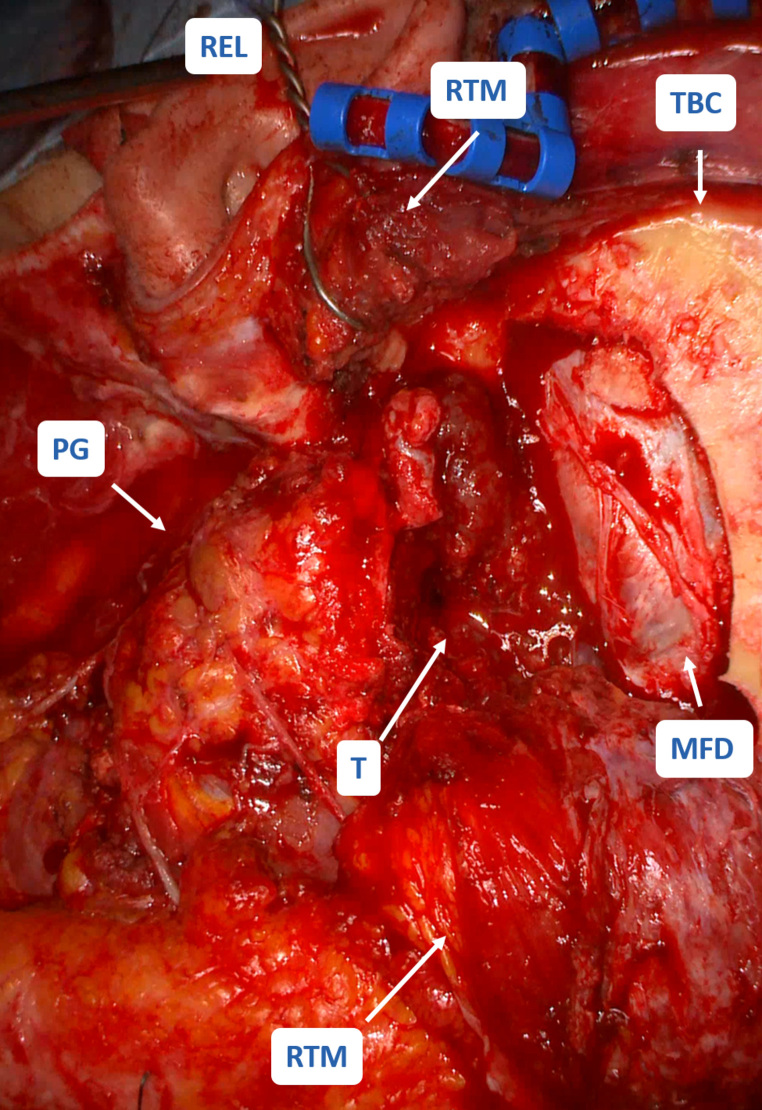
**Operative findings.** Total resection via transparotideal-infratemporal approach. PG: Parotid gland, T: Tumor, TBC: Temporo-basal craniotomy, MFD: Dura of the middle fossa, RTM: Retracted temporalis muscle (split), REL: Retracted ear lobe

We performed a tumor resection via a transparotideal-infratemporal approach, including complete parotidectomy, resection of the zygomatic and the ascending mandibular branch of the facial nerv, evacuation of the infratemporal fossa and radical anterior petrosectomy (Fig. 3). For skull base coverage the temporal bone was covered with galea-periosteum and fibrin glue. The temporo-basal dura was reconstructed by suturing it to galea-periosteum and TachoSil. The sphenoid sinus was occluded with autologous fat and fibrin glue. The bone flap and the resected zygomatic bone were not reinserted due to the uncertainty of the infiltration. The ear was filled with autologous fat and fibrin glue and the ear was closed with a blind-sac closure. Postoperatively, there were no complications despite a complete acute facial palsy which improved progressively. In regular follow-up care no residual tumor has been detected in imaging studies (MRI and CT) within 2 years and 4 months. The initial facial nerve palsy House-Brackmann score (HB) V regressed to HB III.

### Histological features

**Fig. 4:**
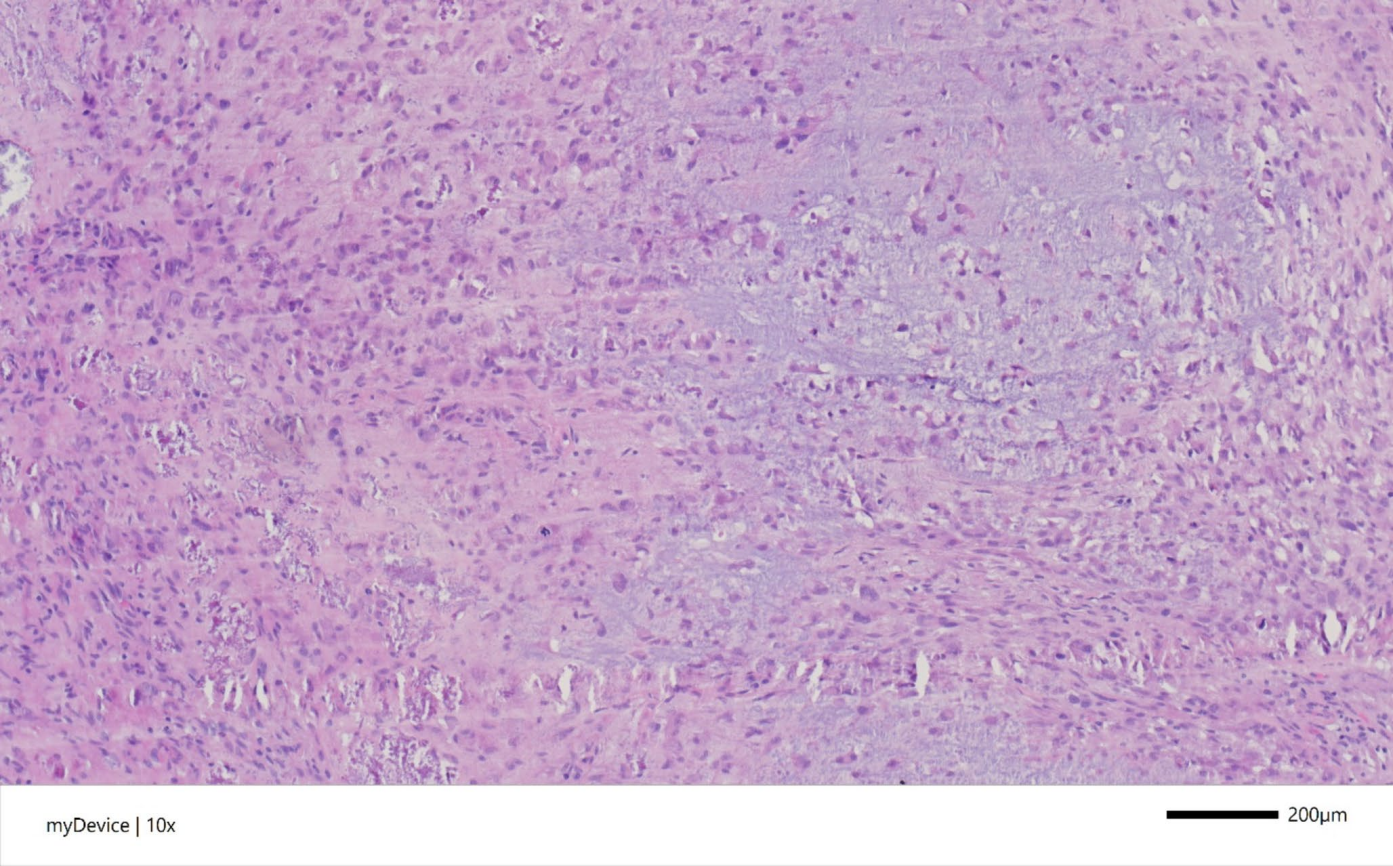
Histological section: Hematoxylin-eosin stain. Augmentation: 100x

Histologically, the tumor was composed of granular variably calcified chondroid matrix with extensive regressive changes and granulation-like tissue reaction associated with calcinosis and crystal deposition **(**Fig. 4**)**. Primitive-looking mesenchymal cells and chondrocytes were embedded within the matrix. There was no epithelial or myoepithelial population or other derivatives of pleomorphic adenoma. Moreover, no histological or molecular (IDH1 or IDH2 mutations) features of chondrosarcoma were present. Molecular analysis of the tumor via the TruSight-RNA-Fusion panel detected a fusion involving FN1::FGFR2, consistent with CCMN.

### Laboratory values

Laboratory testing of calcium phosphate balance revealed slightly decreased calcidiol and calcitriol. The remaining parameters were unremarkable (calcium, anorganic phosphate, ionized calcium, calcium phosphate product, alkaline phosphatase, parathyroid hormone).

## Discussion

Tumors of the lateral skull base and infratemporal fossa are an enormously rare enties with many different histologies including the recently described “calcified chondroid mesenchymal neoplasm” (CCMN) [1–3]. Most patients have symptoms such as a mass, swelling or pain [11]. Because of overlapping radiological and especially histopathological findings, correct diagnosis and managing is still challenging [10, 13]. Other possible diagnoses would be chondroma, tophaceous pseudogout, tenosynovial giant cell tumors, chordomas, chondrosarcoma, chondroblastoma or phosphaturic mesenchymal tumors (PMTs) [5, 9, 10, 13, 15, 16].

Classifying tumors of the skull base preoperatively would be essential, as the entity determines the extent of the surgery to spare the patient unnecessary radical therapy. For example recurrence and survival rates are worse for chordoma than for the rarer chondrosarcoma and benign chondromas do not have to be radically resected [17, 18]. Chordoma and chondrosarcoma cannot be reliably distinguished from each other in CT or MRI scans [17, 19]. The morphologic aspects of the CCMN described here are similar to those of chordoma and chondrosarcoma and a preoperative diagnosis is not possible based on imaging. Because of the infiltrating and destructive growth a malignant diagnoses could have been possible in our case. In contrast to the CCMN in our case the CCMNs in the case report by Kallen et al. mostly had indolent or benign radiologic features. Proposed diagnosis (sorted from frequent to rare) were benign and malignant entities such as soft tissue chondroma, PMT, sarcoma or chondrosarcoma variant, tenosynovial giant cell tumor, ossifying fibromyxoid tumor, osteochondromatous proliferation chondrobastoma or calcifying aponeurotic fibroma. In imaging the reported CCMNs showed a soft tissue mass hyperintense in T2 and hypointense in T1 like in our case as well as internal calcifications [11]. In summary the diagnosis cannot be made on the basis of imaging alone and a histological clarification is required for diagnosis.

There are also overlaps at the molecular level. FN1 fusions with the RTK FGFR1 and FGFR2 can be also observed in soft tissue chondroma and fusions with the RTK FGFR1 in PMTs [16]. PMTs are rare FN1-fusion-associated neoplasms of uncertain histogenesis and typically induce osteomalacia and hypophosphatemia due to FGFR1 induced secretion of Fibroblast growth factor 23 (FGF23) [10, 20]. Due to all these aspects, correct diagnosis is a challenge even when a histological preparation is available and it is possible that CCMNs are underdiagnosed due to the challenging diagnosis.

Due to the challenging diagnostics, preoperative sampling for histological assessment prior to radical resection was not performed. Firstly, imaging led to the strong suspicion of a malignant tumor. Secondly, we expected a complex histological diagnosis combined with the high probability of not being able to determine this reliably in advance by taking a preoperative sample. Of note, even in the first histological assessment, it was not possible to confirm the diagnosis. Only molecular analysis of the tumor via the TruSight-RNA-Fusion panel yielded the final histological diagnosis. In this regard, frozen sections would not allow for a reliable diagnosis intraoperatively. Noteworthy sampling-errors are well-known in enchondromas and atypical chondrogenic tumours due to well differentiated tumour parts [21]. Furthermore due to the recent first description of CCMNs no statement about a possible malignant transformation within the tumor like in other cartilaginous neoplasms had been possible [21].

Recurrence and survival rates of CCMNs cannot be defined precisely since long term results are missing due to the first description of this entity in 2021 [10]. Although there is no documented overtly malignant biological behavior until now [11], no precise statement of the biology and consequently the radio sensitivity can be made due to the low number of cases. There is one reported case of local recurrence after incomplete excision [11]. Because of this and the infiltrating and destructive growth in our case, locally aggressive growth of CCMNs is possible and complete surgical resection is recommended as the treatment of choice including a close follow-up care including imaging.

A partial resection with better facial nerve results in our case is not recommended based on the current state of research. Should CCMNs prove to be benign with little or no risk of malignant degeneration, tumor reduction could be considered as a treatment option.

Further follow-up of CCMNs is needed to understand the biology of CCMNs in terms of local recurrence, metastasis and local growth. In general, further research is needed to better differentiate between the various neoplasms of the skull base preoperatively.

The external therapy attempt with a tympanostomy tube was inadequate. The tumor was subsequently visible on the external CT image and should have been detected.

In contrast to PMTs, no osteomalacia has been described in CCMNs [11]. This is consistent with the unremarkable blood values of the calcium phosphate homeostasis and the clinically irrelevant slightly low calcidiol and calcitriol in our case.

To summarize, the presented tumor is a CCMNs with involvement of the TMJ region and FN1::FGFR2 fusion. At the time of diagnosis, the tumor showed infiltrating and osteo-destructive growth.

## Conclusions

Dealing with tumors of the lateral skull base and infratemporal fossa represents a diagnostic and surgical challenge due to their rarity, diversity and infiltrating behavior, making an interdisciplinary diagnostic work-up and surgical approach mandatory.

Here we describe the extremely rare entity of a “calcified chondroid mesenchymal neoplasm” (CCMN) with an FN1::FGFR2 fusion. Up to now there are only a few reported cases in the medical literature of CCMNs in the TMJ region with FN1 rearrangement within this already extremely rare tumors [10–12].

The biology and consequently the radio sensitivity cannot be defined precisely since long term results are missing due to the first description of this entity in 2021 [10]. As a consequence, surgical resection is recommended as the treatment of choice. Thorough clinical and radiological follow-up is mandatory as local recurrences are to be expected due to the infiltrative behavior. In case of a loco regional recurrence the fusion with FGFR2 may represent a therapeutic option for a targeted therapy on molecular level.
